# Novel Coenzyme Q2 (CoQ2) Mutation in a Pediatric Patient With Primary Steroid-Resistant Nephrotic Syndrome Due to Coenzyme Q10 (CoQ10) Deficiency

**DOI:** 10.7759/cureus.75669

**Published:** 2024-12-13

**Authors:** Shahad Alwazzan, Osama Alnwaihi, Neetha John, Lova Satyanarayana Matsa, Hammad O Alshaya

**Affiliations:** 1 Pediatrics Department, Dr. Sulaiman Al Habib Hospital, Riyadh, SAU; 2 Genomic Precision Diagnostic Department, Igenomix, Dubai, ARE

**Keywords:** coq10 deficiency, coq10 supplementation, coq2 mutations, nephrotic syndrome, steroid-resistant nephrotic syndrome

## Abstract

Coenzyme Q2 (CoQ2) mutations are a group of autosomal recessive mitochondria-linked diseases that result in coenzyme Q10 (CoQ10) deficiency (CoQ10: a cofactor in mitochondrial energy production). Its deficiency leads to multiple systemic clinical presentations; however, isolated steroid-resistant nephrotic syndrome (SRNS) is considerably rare. Multiple genetic mutations have been reported with different ranges of severity and prognosis, with variable responses to CoQ10 supplementation. This case report describes a boy with CoQ10 deficiency due to a novel homozygous variation in the CoQ2 gene, c.1112T>A, p.(Leu371Gln). The patient presented with isolated SRNS, and oral supplementation of CoQ10 resulted in remission.

## Introduction

Nephrotic syndrome is a common childhood disorder. Most cases respond well to steroids; however, only about 20% fail to respond to steroid therapy. In about 30% of steroid-resistant patients, inherited genetic mutations can be detected [1]. Mutations that lead to coenzyme Q10 (CoQ10) deficiency are a rare but treatable form of steroid-resistant nephrotic syndrome (SRNS) and constitute about 2% of all causes of SRNS [2].

Primary CoQ10 deficiency is an autosomal recessive mitochondrial disorder caused by a mutation in one of the genes responsible for the biosynthesis of CoQ10, including coenzyme Q2 (CoQ2). Its manifestations are multisystemic and rarely limited to the kidneys [2]. The CoQ2 gene is located on the q arm of chromosome 4, corresponding to 4q21.23. CoQ10 supplementation has shown a significant improvement in proteinuria, edema, and general patient conditions [3]. We report a pediatric patient with SRNS who was found to have CoQ10 deficiency due to a novel CoQ2 gene mutation and showed a complete response to oral CoQ10 supplementation.

## Case presentation

**Figure 1:**
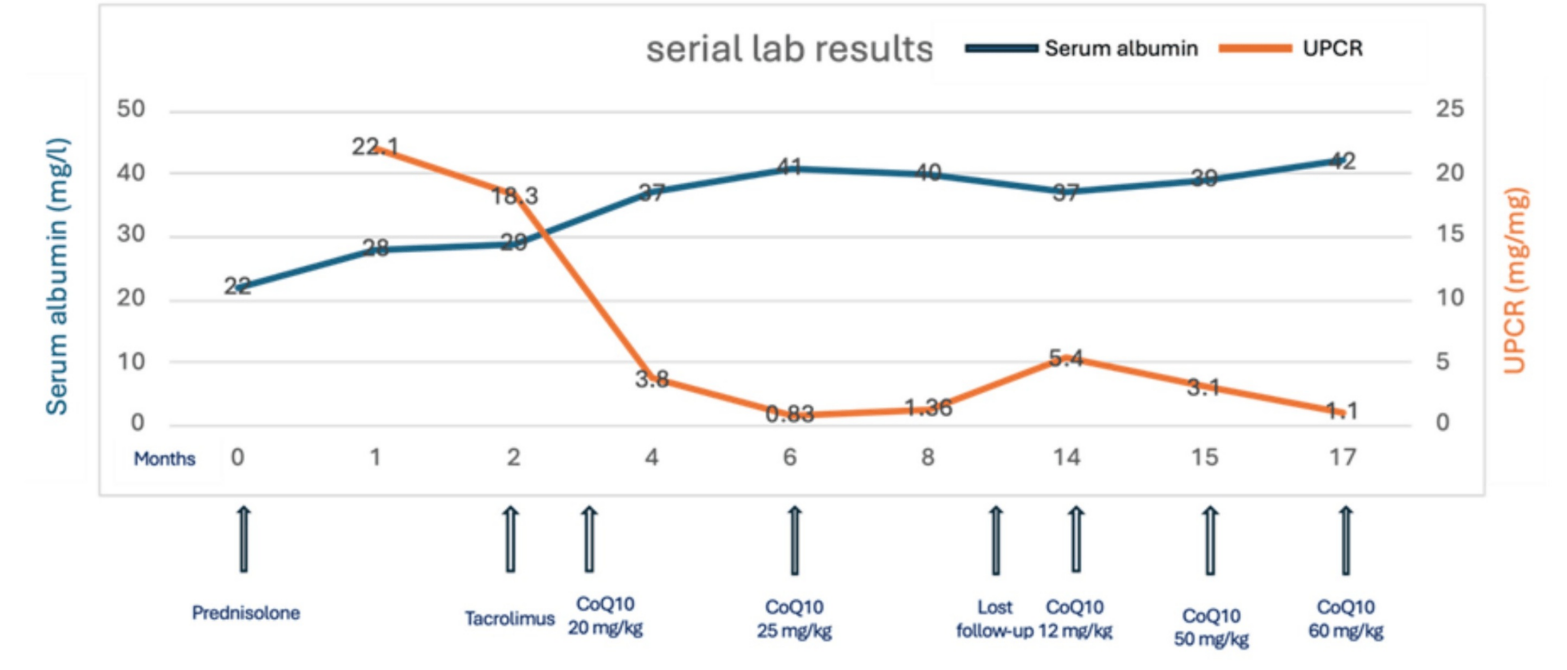
Serial laboratory results of serum albumin and UPCR showing the effect of medications and proper CoQ10 supplementation UPCR: urine protein/creatinine ratio; CoQ10: coenzyme Q10

A two-year-old boy from Syria was evaluated at the age of one for bilateral foot swelling and the presence of frothy urine. His urine was positive for protein 3+, urine protein/creatinine ratio (UPCR) was 22.1 mg/mg (normal <0.2), and serum albumin was 22 g/L (normal range 38-54). He was started on daily oral prednisolone 2 mg/kg/day. Unfortunately, he did not show a response after completing six weeks of prednisolone therapy followed by methylprednisolone intravenous pulse therapy (600 mg/m^2^) for three days. He showed partial improvement in serum albumin up to 32 g/L; however, UPCR remained high at 18.3. A whole exome sequencing test was done, but the parents did not agree on a kidney biopsy due to fiscal limitations. He was started on tacrolimus at a dose of 0.1 mg/kg/day guided by level until the results of genetic testing were received. Genetic results showed a homozygous variation in the CoQ2 gene, NM_015697.7:c.1112T>A, p.(Leu371Gln). Both tacrolimus and prednisolone were weaned off, and the patient was started on CoQ10 oral supplementation with a dose of 20 mg/kg/day, which was increased progressively to 60 mg/kg/day. His serum albumin level normalized, and the urine dipstick showed negative-to-trace for protein, with a significant reduction in UPCR, as illustrated in Table 1 and Figure 1.

**Table 1:** Serial laboratory results of serum albumin and UPCR from the onset of diagnosis and start of management UPCR: urine protein/creatinine ratio

Months (from the onset of diagnosis and start of management)	Serum albumin (normal range: 38-54 g/L)	UPCR (normal ratio <0.2)
1	28	22.16
2	29	18.3
4	37	3.8
6	41	0.83
8	40	1.36
14	37	5.4
15	39	3.1
17	42	1.1

Molecular findings

The genetic study of the patient showed that he was homozygous for a variant of uncertain significance, NM_015697.9:c.1112T>A, p.(Leu371Gln), in the CoQ2 gene that replaces the amino acid Leucine with glutamine at codon 371. Pathogenic variations in the CoQ2 gene are associated with CoQ10 deficiency, primarily one with an autosomal recessive mode of inheritance. This variant was classified as a variant of uncertain significance according to the American College of Medical Genetics and Genomics guidelines and the Association of Clinical Genomic Science guidelines [4,5]. Target mutation testing was performed for the identified variant through minisequencing or primer extension technique (3500XL, Applied Biosystem, Carlsbad, CA), which involves the use of a single base extension primer whose 3' end is located next to the base preceding the variant of our interest. Primers used for testing were represented in Table 2. The target mutation testing revealed that the parents and unaffected sister were heterozygous for this CoQ2 gene variant, as shown in Table 3. Since the patient's clinical details are compatible with the disease, and based on the segregation analysis, this CoQ2 variant can be considered as likely pathogenic.

**Table 2:** Primers used for target testing MS: minisequencing

Variant-specific primers	MS primers
Forward: ACCCAAAGTCCAGACTAGGTAT	MS-forward: CTCTCTTGCAGATTTACACTC
Reverse: ATTAGTCCCAGTGTTCGGTTG	MS-reverse: ATCCTCAGGTCTGTGGATGTCT

**Table 3:** Results of target mutation testing of parents and unaffected sister CoQ2: coenzyme Q2

CoQ2 variant	Proband	Affection status	Result
NM_015697.9:c.1112T>A, p.(Leu371Gln)	Index	Affected	Homozygous
	Mother	Unaffected	Heterozygous
	Father	Unaffected	Heterozygous
	Sister	Unaffected	Heterozygous

## Discussion

CoQ10 is a crucial component of mitochondrial energy production and serves as a vital antioxidant, protecting cells from oxidative damage caused by free radicals. Mutations in the CoQ2 gene disrupt CoQ10 synthesis, leading to a deficiency of this essential molecule. This deficiency can have widespread effects, resulting in various clinical manifestations.

The impact of CoQ2 gene mutations includes mitochondrial encephalomyopathy and nephropathy. Mitochondrial encephalomyopathy is a condition that involves dysfunction in both the brain and muscles due to impaired mitochondrial energy production. Patients may experience neurological symptoms such as ataxia, seizures, developmental delays, muscle weakness, and pain. Nephropathy is a condition that involves CoQ10 deficiency, which can also affect kidney function, potentially leading to nephropathy, which may present as kidney dysfunction or progressive kidney failure. The broad range of symptoms associated with CoQ2 mutations highlights the essential role of CoQ10 in maintaining cellular health and the complex nature of disorders arising from its deficiency [6].

The mode of inheritance for mutations in the CoQ2 gene is autosomal recessive. Homozygosity of these mutations is associated with a more severe form of the disease and an earlier onset of clinical manifestations [6]. In some cases, such as the one reported in our patient, isolated SRNS can occur as the primary manifestation of CoQ10 deficiency without the additional extrarenal symptoms often seen in other cases. This presentation aligns with previously published reports where SRNS was the predominant symptom due to CoQ2 mutations, highlighting the variability in how the disease can manifest [7].

The CoQ2 gene is pivotal for synthesizing CoQ10, a molecule essential for mitochondrial energy production and overall cellular function. Missense mutations in CoQ2, which was found in our patient's test, as well as many previously reported cases, have been linked to various diseases due to their impact on the enzyme's function, which, in turn, disrupts CoQ10 production and leads to clinical symptoms. In the case of our patient, the novel mutation identified in the CoQ2 gene has not been previously documented in the literature. This discovery is significant because it broadens the range of known mutations associated with CoQ10 deficiency. Identifying new mutations helps to enhance our understanding of the genetic basis of CoQ10 deficiency, which can improve diagnostic accuracy and potentially guide more effective treatment strategies [6].

Although the administration of oral CoQ10 supplementation is highly effective in these cases and should be initiated early in the disease to be effective, it was illustrated in research done on 303 patients with primary CoQ10 deficiency due to different gene mutations. Seven of them were due to CoQ2 mutation, and all patients received CoQ10 supplement, but only one patient was reported to be a responder to the supplementation [3-8].

Fortunately, in our patient, therapy was initiated relatively early, so we were able to prevent permanent damage that might have happened to the kidneys or other systems. The appropriate dose of CoQ10 is not well defined; however, in our case, it seemed to be around 50-60 mg/kg/day in three divided doses. A careful and close follow-up of these young patients is very important as they grow fast, and one needs to adjust the dose accordingly to prevent relapse similar to what happened in our patient. In addition, we tried dividing the dose to Q12h, but again he started to expel more protein in urine. Although we managed to keep his serum albumin in the normal range and significantly reduce proteinuria, with negative-to-trace for protein in urine dipstick, we were unable to make UPCR back to the normal range (the lowest UPCR achieved was 1.1 mg/mg) despite using a different formulation of CoQ10. No side effects of the enzyme have been noticed so far over the past 14 months.

## Conclusions

In conclusion, primary CoQ10 deficiency, being a treatable condition, should be ruled out promptly to avoid delays in effective management. Given that CoQ10 supplementation is generally safe and relatively inexpensive, initiating therapy could be beneficial, especially for young children with SRNS, while awaiting genetic test results. This proactive approach can potentially mitigate symptoms and improve outcomes, ensuring that patients receive timely intervention based on their clinical presentation and suspected diagnosis.
